# Umbilical cord blood derived cell expansion: a potential neuroprotective therapy

**DOI:** 10.1186/s13287-024-03830-0

**Published:** 2024-07-29

**Authors:** Tayla R. Penny, Graham Jenkin, Suzanne L. Miller, Courtney A. McDonald

**Affiliations:** 1 The Ritchie Centre, Hudson Institute of Medical Research, Clayton, VIC Australia; 2 Department of Obstetrics and Gynaecology, Monash University, Clayton, VIC Australia

**Keywords:** Umbilical cord blood, Haematopoietic stem cell, Expansion, Neuroprotective

## Abstract

Umbilical cord blood (UCB) is a rich source of beneficial stem and progenitor cells with known angiogenic, neuroregenerative and immune-modulatory properties. Preclinical studies have highlighted the benefit of UCB for a broad range of conditions including haematological conditions, metabolic disorders and neurological conditions, however clinical translation of UCB therapies is lacking. One barrier for clinical translation is inadequate cell numbers in some samples meaning that often a therapeutic dose cannot be achieved. This is particularly important when treating adults or when administering repeat doses of cells. To overcome this, UCB cell expansion is being explored to increase cell numbers. The current focus of UCB cell expansion is CD34+ haematopoietic stem cells (HSCs) for which the main application is treatment of haematological conditions. Currently there are 36 registered clinical trials that are examining the efficacy of expanded UCB cells with 31 of these being for haematological malignancies. Early data from these trials suggest that expanded UCB cells are a safe and feasible treatment option and show greater engraftment potential than unexpanded UCB. Outside of the haematology research space, expanded UCB has been trialled as a therapy in only two preclinical studies, one for spinal cord injury and one for hind limb ischemia. Proteomic analysis of expanded UCB cells in these studies showed that the cells were neuroprotective, anti-inflammatory and angiogenic. These findings are also supported by in vitro studies where expanded UCB CD34+ cells showed increased gene expression of neurotrophic and angiogenic factors compared to unexpanded CD34+ cells. Preclinical evidence demonstrates that unexpanded CD34+ cells are a promising therapy for neurological conditions where they have been shown to improve multiple indices of injury in rodent models of stroke, Parkinson’s disease and neonatal hypoxic ischemic brain injury. This review will highlight the current application of expanded UCB derived HSCs in transplant medicine, and also explore the potential use of expanded HSCs as a therapy for neurological conditions. It is proposed that expanded UCB derived CD34+ cells are an appropriate cellular therapy for a range of neurological conditions in children and adults.

## Introduction

Umbilical cord blood (UCB) is a well-studied source of stem cells and the first UCB cell transplant was performed in 1988 to treat Fanconi's anaemia [1]. Since then, > 40,000 UCB cell transplants have been performed where the primary clinical application of UCB cells is for haematological conditions, particularly blood cancers [2–5]. The use of UCB derived cells in transplants for haematological conditions has significant advantages over other sources of cells due to their ability to engraft and repopulate the immune system as seen by neutrophil and platelet recovery [6].

Preclinical studies and clinical trials have also been conducted to examine the efficacy of UCB in a regenerative medicine capacity as a therapy for multiple non-haematological conditions including, diabetes [7–10], heart failure [11, 12], cerebral palsy [13–17], stroke [18–20] and spinal cord injury [21–23]. The benefits of UCB in regenerative medicine are thought to be attributed to the presence of a heterogenous population of naïve stem and progenitor cells and potent immunosuppressive cells which are present in varying concentrations in cord blood. Specifically, the mononuclear cell (MNC) population found in UCB is composed of a variety of cells, including stem and progenitor cells (Table 1). The presence of these cell types is not unique to UCB as they are found throughout the body, however it is thought that the combination of these cell types and their naivety contributes to their beneficial effect. In addition, they convey a reduced risk of graft versus host disease (GVHD) and rejection when compared to adult sources of cells [29].

**Table 1:** Major cellular constituents of UCB and examples of cell surface markers commonly used to identify MNCs in UCB

Cell type	Proportion of MNCs (%)	Positive for cell surface markers	Negative for cell surface markers	References
Haematopoietic stem cells (HSCs)	0.02–1.43	CD34^+^ CD45^+^ CD90^+^	CD38^−^ CD45RA^−^	[24, 30]
Mesenchymal stem/stromal cells (MSCs)	< 0.01	CD105^+^ CD90^+^ CD73^+^ CD44^+^ STRO-1^+^	CD34^−^ CD45^−^ HLA-DR^−^ CD11b^−^ CD14^−^	[25, 31]
Regulatory T- cells (Tregs)	1–5	CD3^+^ CD4^+^ CD25^+^ FoxP3^+^	CD34^−^	[26, 32]
Monocyte derived suppressor cells (MDSCs)	5	CD14^+^ CD11b^+^ CD16^±^ CD66b^±^	HLA-DR^low/−^	[27, 33]
Endothelial progenitor cells (EPCs)	0.2–1	CD34^+^ CD133^+^ VEGFR2^+^	CD45^−^ CD31^−^	[28, 34]
Lymphocytes	15–51	CD3^+^ CD4^+^ CD8^+^ CD19^+^	CD34^−^	[25, 35]
Dendritic cells (DCs)	0.01–1.6	CD11C^+^ CD45^+^ MHC-II^+^	CD3^−^ CD56^−^ CD19^−^ CD20^−^ CD16^−^	[36]

Besides use of the total mononuclear cell fraction, studies have investigated the therapeutic potential of specific cell types found within UCB, particularly HSCs and MSCs [13]. In regenerative medicine applications, UCB derived MNCs are thought to act via paracrine effects and by promoting an endogenous response to injury. As such, UCB derived MNCs have been broadly shown to promote angiogenic and neuroregenerative responses as well as having anti-inflammatory and immune-modulatory effects [16, 37]. In addition, MNCs have been shown to improve functional deficits following neurological injury [38].

To achieve a therapeutically effective dose for engraftment and reconstitution of the haematopoietic stem cell niche, the availability of sufficient cells in a single unit of UCB for clinical trials, and now clinically for haematological conditions, has previously limited the use of UCB in autologous and allogeneic matched transplantation to children and adolescents. Transfusion of multiple units of allogeneic UCB are now, increasingly, being used to ensure an adequate therapeutic dose is achieved particularly in adults [39]. This further increases the risk of GVHD and it is often difficult to find multiple human leukocyte antigen (HLA) matched donors, particularly for people of non-Caucasian origin [40, 41]. To address this potential barrier, stem cell expansion was developed as an alternative strategy to increase total cell number for transplantation. However, the heterogeneity of cell populations within UCB necessitates different expansion conditions that require individualised optimisation for each cell type. Expansion studies to date have predominantly focused on haematopoietic stem and progenitor cells (HSPCs) for expansion, as these cells are most relevant to transplantation medicine where haematological malignancies are the primary focus [42]. HSC expansion has been well studied, and the methods used to achieve expansion are varied and result in different rates of expansion (ranging from 35 fold [43] to 1594 fold expansion [44]) and differentiation into other cell types. Currently there are 36 registered clinical trials that are investigating the therapeutic potential of expanded UCB cells, with 73% of these trials using expanded HSCs. These clinical trials span treatment of various conditions including haematological conditions and metabolic disorders. The variety of methods by which HSCs are expanded, and their use in clinical trials are summarised below. In an exciting very recent development, the Gamida-Cell Ltd UCB expanded cell product, “Omisurge”, was granted market approval from the FDA [45].

Unexpanded UCB and HSCs have been shown to be effective as a potential therapy for multiple neurological conditions, including perinatal brain injury [14, 37, 46, 47] and subsequently cerebral palsy [48, 49], ischemic stroke [18, 50], and in adults for Parkinson’s disease [51]. Although at present HSC expansion is predominantly used in transplant medicine where the goal of therapy is engraftment and reconstitution of the immune system, it is becoming apparent that there are multiple potential benefits that lie in regenerative medicine applications, particularly where engraftment is not required to elicit a therapeutic response. Outside of haematological studies, there are no clinical and very few preclinical studies that have investigated the use of expanded HSCs as a therapy. Currently, this therapy has only been trialled in the setting of spinal cord injury [52] and hind limb ischemia [53] where expanded HSCs were shown to promote tissue repair and functional improvements.

To meet this perceived increasing demand for UCB derived cells repurposing the use of expanded UCB derived cells for regenerative medicine applications will, in our opinion, be essential. This review will discuss the current use of expanded HSCs in transplantation medicine and highlight the potential of expanded HSCs for regenerative medicine purposes, specifically in the context of neurological conditions. It is proposed that expanded UCB derived HSCs will be a safe and efficacious treatment for a range of brain injuries observed in both adults and children.

## Haematopoietic stem cells

**Fig. 1:**
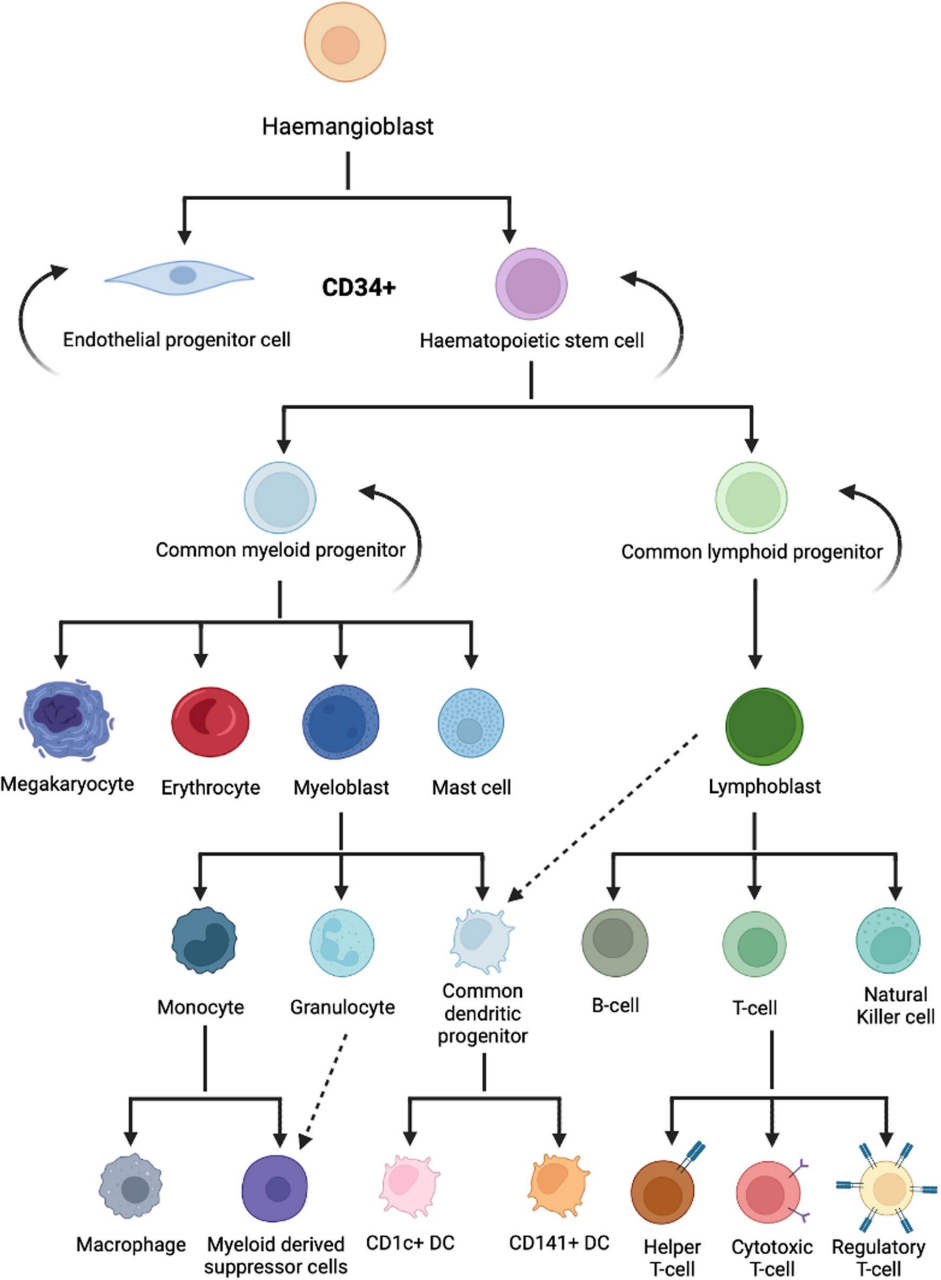
Haematopoietic lineage of differentiation. This schematic demonstrates the current understanding of the differentiation potential of haematopoietic stem cells following early differentiation into either a common myeloid or lymphoid progenitor cell. (Created with BioRender.com)

Stem cell therapies are now established in clinical practice in transplantation and engraftment applications particularly as a treatment option for individuals suffering from haematological malignancies such as leukemias and lymphomas [54]. More recently there has been a focus on a plethora of regenerative medicine potential applications, although most of these are still being investigated in preclinical studies and in the clinical trial phase of use. HSCs have been the focus of cell therapy research since the first bone marrow transplant in 1956 and have principally been used for haematological disorders such as leukemia [55]. HSCs are multipotent cells that can differentiate into cells of the blood lineage- broadly, red blood cells, white blood cells and platelets [56]. The cell surface antigen cluster of differentiation 34 (CD34) is a marker of early, multipotent haematopoietic cells and is often used clinically to quantify the number of HSCs available for use in transplantation [57]. Upon differentiation, haematopoietic cells lose their CD34 marker and become CD34 negative [58]. HSCs can differentiate down the myeloid or lymphoid lineage to give rise to all haematopoietic cells [59] (Fig. 1), which allows for complete immune reconstitution when used as a treatment for haematological conditions [60].

### Advantages and disadvantages of umbilical cord blood derived HSCs

Bone marrow (BM) and mobilised peripheral blood (MPB) are widely accepted to be the most common source of cells used in transplant applications, specifically HSCs and MSCs are the cells commonly isolated from these sources [40, 61–63]. Despite this, BM and MPB have inherent restrictions associated with them. They require painful/invasive procedures for collection and are associated with a high risk of adverse events [39].

More recent studies have focused on UCB as an alternative source of HSCs for cellular therapies as this is associated with less restrictions [64] (Table 2).

**Table 2:** Advantages and disadvantages of HSC sources

Source	Advantages	Disadvantages
Bone Marrow	Standard source of stem cells for transfusion [40] Higher CD34+ cell numbers than UCB [39]	Higher risk of GVHD Invasive procedure to collect Stringent HLA requirements (8/8) [65]
Mobilised Peripheral Blood	Less invasive than BM collection [39] Highest CD4 + cell numbers [39] Fast engraftment	Higher risk of GVHD Cells need to be mobilised with G-CSF Stringent HLA requirements (8/8) [65] High number of T-cells [39]
Umbilical cord blood	No ethical challenges Non-invasive collection procedure Can tolerate up to 2 HLA mismatches [67] Naïve cells Low number of T-cells [39] Lower risk of GVHD [39] Lower risk of rejection	Lowest CD34+ cell numbers [24] Slower engraftment [39]

*GVHD* Graft versus host disease, *HLA* Human Leukocyte Antigen, *BM* Bone marrow, *G-CSF* Granulocyte colony stimulating factor

The use of MPB as a source of HSCs was implemented as an alternative to BM as it involves a less painful procedure and has a lower risk associated with collection. MPB also has a higher CD34+ concentration compared to BM and is associated with a lower risk of GVHD upon transplantation [39]. One of the main restrictions of both BM and MPB derived cells is the need for extensive HLA matching. For transplantation of adult BM and MPB, the HLA matching criteria for unrelated donors must be 7/8 or 8/8 matching loci and for fully matched siblings, the requirement is a 6/6 match [65]. This is not always feasible, as there is often a lack of suitably matched HLA donors, particularly for ethnic minorities [40].

Alternatively, UCB derived HSCs have low ethical considerations, are easy to collect and their collection poses no risk to the donor, since cord blood is collected after birth. UCB derived HSCs may in fact be a preferred source of HSCs for therapeutic use due to their relative naivety and highly proliferative nature [24, 66]. Due to the presence of immature immune cells in UCB, HLA matching can be less stringent than with other sources, as multiple antigen mismatches can be tolerated whilst still reducing the risk of GVHD upon transplantation [39, 40]. Specifically, transplantation of donor UCB can tolerate HLA mismatches at up to two loci, thus have a matching criterion of 4/6 to 6/6 [67]. Despite these advantages, UCB has the lowest concentration of CD34+ cells, compared to BM and MPB, with CD34+ cells only comprising 0.02–1.43% of all UCB mononuclear cells [24], and UCB derived cells also show slower engraftment compared to other sources [39]. As such, multiple UCB unit infusions are currently used to increase therapeutic potential, but this poses the difficulty of finding multiple HLA matched donors, which in turn can contribute to a higher risk of GVHD [39]. Due to the relatively low CD34+ concentration in UCB, cell expansion is being investigated to increase cell numbers available for infusion. This allows for treatment with multiple doses of autologous cells, as well as increasing cell numbers available for allogeneic donation, banking and potential use.

## Umbilical cord blood expansion

In order to increase the number of cells available for transplantation, methods of expanding UCB derived stem cells have been investigated. These expansion studies have predominantly focused on expanding the haematopoietic fraction of UCB as an emerging therapy for haematological malignancies.

### Expansion strategies

Initial expansion studies involved culturing UCB derived HSCs in a cocktail of haematopoietic growth factors including thrombopoietin (TPO), Fms-like tyrosine kinase 3 ligand (Flt3), Interleukin 6 (IL-6), Interleukin 3 (IL-3) and stem cell factor (SCF) [68, 69]. Whilst these factors successfully induced haematopoietic cell proliferation, the cell yield was low and with significant differentiation of the native cells, restricting the number of HSCs available for transfusion [70]. As such, novel methods are being investigated to enhance the rate of UCB derived HSC expansion, whilst promoting symmetrical cell division, rather than differentiating cell populations [71]. These current expansion strategies have been well documented [29, 72, 73], and thus will only be briefly discussed here.

#### Aryl hydrocarbon antagonists

The Aryl hydrocarbon Receptor (AhR) antagonist Stem Regninin-1 (SR-1), when combined with haematopoietic growth factors, has been shown to successfully expand CD34+ cells in vitro via inhibition of aryl hydrogen receptor signalling [70]. Culture with SR-1 has been reported to increase the number of MPB CD34+ cells by 1118-fold after a 3-week culture period and promoted expansion of UCB CD34+ cells by 17,100-fold increase following a 5-week culture period. SR-1 also reduces CD34 differentiation, where following 5-weeks of culture with SR-1, the expanded population comprised of 66–76% CD34+ cells, in comparison to controls (no SR-1; 14–31% CD34+ cells) [70]. To date there have been three phase I/II clinical trials using an SR-1 expanded UCB CD34+ cell product known as MGTA-456 (previously HSC835) for leukemia and lymphomas, as well as inherited metabolic disorders where engraftment and neutrophil recovery were the primary outcomes. Results from these trials show that expansion using SR-1 resulted in an average of 330–491-fold increase in CD34+ cells [74–76], and that infusion of SR-1 expanded cells was safe and feasible.

#### Pyrimidoindole derivatives

Pyrimidoindole derivative UM729 was identified as a low molecular weight compound that had the ability to promote expansion of CD34+ cells by enriching a population of long-term HSCs. The related molecule, UM171, a synthetic analogue, was shown to be 10–20 times more potent than UM729, thus further studies were conducted using UM171 [77]. Unlike SR-1, UM171 does not supress the AhR pathway, but instead is thought to target the transcriptional corepressor complex CoREST, comprising of lysine-specific histone demethylase 1A (LSD1A), histone deacytylase 1 (HDAC1) and rest corepressor 1 (RCOR1), which is known to inhibit HSC self-renewal. Further, degradation of LSD1 and RCOR1 promotes in vitro expansion of human HSCs similarly to UM171 [78]. Expansion with UM171 in combination with SR-1 has been shown to increase CD34+CD45+ cells 1120-fold after 14 days, with CD34+ cells making up ~ 80% of the expanded cell population [79]. UM171 expanded CD34+ cells have also been implemented in clinical trials, with 6 trials currently registered (Table 3). A trial by Cohen et al. demonstrated a 35-fold increase in cell number after 7 days of expansion and demonstrated the safety and feasibility of treatment with UM171 expanded CD34 cells for haematological transplantation [43].

**Table 3:** Clinical trials using expanded UCB cell therapies

Status	Study name	Sponsor	Study type	Condition	Primary outcome	Cell therapy		Additional drugs or compounds	Age	Patients enrolled (or target)	Clinical trial ID
						Expansion strategy	Expanded cell dose				
Completed	VPA Expanded UCB Transplantation for Treatment of Patients With Hematological Malignancies	Icahn School of Medicine at Mount Sinai, USA	Open label single arm	Haematological conditions	Safety and engraftment	VPA	N/S	Immunosuppression, unmanipulated UCB	18–65 years	7	NCT03885947
Recruiting	US Study of UM171-Expanded CB in Patients With High Risk Leukemia/Myelodysplasia	ExCellThera, Canada	Open label single arm	Leukemias/ myelodysplastic Syndrome	Adverse events & relapse-free survival	UM171	0.25–5 × 10^6^ cells/kg	Immunosuppression, unexpanded UCB CD34- infusion	18–65 years	20	NCT04103879
Recruiting	Unrelated Umbilical Cord Blood Transplantation for Severe Aplastic Anemia and Hypo-plastic MDS Using CordIn(TM), Umbilical Cord Blood-Derived Ex Vivo Expanded Stem and Progenitor Cells to Expedite Engraftment and Improve Transplant Outcome	National Heart, Lung, and Blood Institute (NHLBI), USA	Open label single arm	Severe aplastic anaemia and myelodysplastic syndrome	Engraftment	CordIn™	N/S	Unexpanded CD133- fraction	4–75 years	37	NCT03173937
Completed	Umbilical Cord Transplantation for the Elderly Population	Loyola University, USA	Open label parallel arm	Leukemias/ lymphomas	Efficacy	StemEx®	N/S	Unmanipulated UCB	55–73 years	18	NCT01484470
Completed	Umbilical Cord Blood Transplant with Added Sugar and Chemotherapy and Radiation Therapy in Treating Patients With Leukemia or Lymphoma	M.D. Anderson Cancer Center, USA	Non-randomized open label parallel arm	Leukemias/ lymphomas	Safety, feasibility & engraftment	Fucosylated expanded UCB- mesenchymal progenitor cell co-culture	N/S	Chemotherapy, total body irradiation, GVHD prophylaxis	12–65 years	6	NCT03096782
Completed	Umbilical Cord Blood Transplant for Hematological Malignancies (UCB) [80]	University of Pennsylvania, USA	Open label single arm	Leukemias/ lymphomas	Dose limiting toxicity	Expanded UCB T-cells	0.1–400 × 10^6^ cells/kg	Myeloablative pre-conditioning, unmanipulated UCB	21–50 years	5	NCT00891592
Completed	Umbilical Cord Blood NK Cells, Rituximab, High-Dose Chemotherapy, and Stem Cell Transplant in Treating Patients With Recurrent or Refractory B-Cell Non-Hodgkin's Lymphoma [81]	M.D. Anderson Cancer Center, USA	Open label single arm	B-cell Non-Hodgkin's lymphoma	Survival	UCB expanded NK cells	1 ×10^8^ cells/kg	Chemotherapy	15–70 years	22	NCT03019640
Recruiting	UM171 Expanded Cord Blood In Patients With High-Risk Acute Leukemia/Myelodysplasia	Ciusss de L'Est de l'Île de Montréal, Canada	Open label single arm	Leukemias/ myelodysplastic Syndrome	Survival & relapse free survival	UM171	N/S	Chemotherapy, immunosuppression, unexpanded UCB CD34- infusion	18–70 years	20	NCT03913026
Completed	Trial of AB-110 in Adults With Hematologic Malignancies Undergoing Cord Blood Transplantation	Angiocrine Bioscience, USA	Open label single arm	Leukemia/ myelodysplastic syndromes	Occurrence of adverse events & engraftment	AB-110 (endothelial cell co-culture)	N/S	Unmanipulated UCB	18–60 years	10	NCT03483324
Active, not recruiting	Trial Evaluating MGTA-456 in Patients With High-Risk Malignancy	Masonic Cancer Center, University of Minnesota, USA	Open label single arm	Leukemias/ lymphomas	Neutrophil recovery	MGTA-456 (SR-1)	> 10 × 10^6^ cells/kg	Chemotherapy, immunosuppression	< 55 years	22	NCT03674411
Completed	Transplantation of NiCord®, Umbilical Cord Blood-derived Ex Vivo Expanded Cells, in Patients With HM [82]	Gamida Cell ltd, USA	Open label single arm	Haematological malignancies	Engraftment	NiCord®	1.4–14.9 × 10^6^ cells/kg	N/S	12–65 years	36	NCT01816230
Completed	Transplantation of Ex-vivo Expanded Cord Blood Stems Cells (GRAPA) [83]	University Hospital, Bordeaux, France	Open label single arm	Haematological malignancies	Granulocyte number	Expanded UCB CD34+ cells	N/S	UCB CD34- infusion	18–65 years	16	NCT01034449
Active, not recruiting	Stem Cell Transplantation With NiCord® (Omidubicel) vs Standard Umbilical Cord Blood in Patients With Leukemia, Lymphoma, and Myelodysplastic Syndrome (MDS) [84]	Gamida Cell ltd, USA	Randomized open label parallel arm	Leukemia/ lymphoma/ myelodysplastic syndrome	Neutrophil engraftment	NiCord®	21.1–47.6 × 10^6^ cells/kg	Unmanipulated UCB, non-cultured UCB fraction	12–65 years	124	NCT02730299
Completed	Safety and Tolerability of HSC835 in Patients With Hematological Malignancies [74]	Novartis Pharmaceuticals, USA	Open label single arm	Leukemias/ lymphomas	Toxicity, graft failure & relapse	HSC835 (SR-1)	30–270 × 10^6^ cells/kg	Unexpanded UCB and CD34- fraction	10–55 years	27	NCT01474681
Recruiting	Safety and Efficacy of Umbilical Cord Blood Regulatory T Cells Plus Liraglutide on Autoimmune Diabetes	Second Xiangya Hospital of Central South University, China	Randomized open label parallel arm	Type 1 diabetes	Safety	Expanded UCB derived Tregs	2 × 10^6^ cells/kg	Liraglutide (diabetic medication) OR insulin	> 18 years	40	NCT03011021
Completed	Randomized Double Cord Blood Transplant Study	M.D. Anderson Cancer Center, USA	Randomized open label parallel arm	Leukemias/ lymphomas	Engraftment	NiCord®	N/S	Chemotherapy, unmanipulated UCB	1 m-80 years	110	NCT00067002
Completed	Pilot Study Evaluating Safety & Efficacy of DCBT: NiCord® & UNM CBU to Patients With Hematological Malignancies [85]	Gamida Cell ltd	Open label single arm	Leukemia/ lymphoma/ myelodysplastic syndrome	Toxicity & engraftment	NiCord®	0.9–18.3 × 10^6^ cells/kg	Unmanipulated UCB	8–65 years	12	NCT01221857
Completed	P3 Study of Umbilical Cord Blood Cells Expanded With MPCs for Transplantation in Patients With Hematologic Malignancies	Mesoblast, Ltd	Randomized open label parallel arm	Haematological malignancies	Engraftment	Expanded UCB- mesenchymal progenitor cells	N/S	Unmanipulated UCB	< 65 years	49	NCT01854567
Recruiting	Intrathecal Administration of DUOC-01 in Adults With Primary Progressive Multiple Sclerosis (DUOC for MS)	Duke University, USA	Open label single arm	Multiple sclerosis	Safety	DUOC-01 (oligodendrocyte like cells)	10–50 × 10^6^ cells/kg	Hydrocortisone	18–65 years	20	NCT04943289
Recruiting	Infusion of Expanded Cord Blood Cells in Addition to Single Cord Blood Transplant in Treating Patients With Acute Leukemia, Chronic Myeloid Leukemia, or Myelodysplastic Syndromes	Fred Hutchinson Cancer Research Center, USA	Open label single arm	Leukemia	Safety & engraftment	NLA101	N/S	Chemotherapy, immunosuppression, unmanipulated UCB	18–65 years	15	NCT03399773
Completed	Infusion of Off-the-Shelf Expanded Cord Blood Cells to Augment Cord Blood Transplant in Patients With Hematologic Malignancies [86]	Nohla Therapeutics, Inc., USA	Open label single arm	Haematological malignancies	Safety & engraftment	NLA101	3.1–11.6 × 10^6^ cells/kg	Chemotherapy, immunosuppression, unmanipulated UCB	6 m-45 years	15	NCT01175785
Completed	Expanded Cord Blood in Patients in Need of an Allogeneic Stem Cell Transplant [43]	Maisonneuve-Rosemont Hospital, Canada	Non-randomized open label factorial assignment	Haematological malignancies	Safety	UM171	0.025–0.49 × 10^6^ cells/kg	Myeloablative pre-conditioning	3–64 years	25	NCT02668315
Completed	Efficacy and Safety Study of StemEx®, to Treat Subjects With High Risk Hematologic Malignancies, Following Myeloablative Therapy (ExCell) [87]	Gamida Cell ltd, USA	Open label single arm	Leukemia/ lymphoma/ myelodysplastic syndrome	Safety	StemEx®	9.7 × 10^5^ cells/kg	Unmanipulated UCB	12–55 years	101	NCT00469729
Active, not recruiting	ECT-001 (UM171) Expanded Cord Blood Transplant to Treat High-risk Multiple Myeloma	ExCellThera inc., Canada	Open label single arm	Multiple myeloma	Safety & feasibility	UM171	N/S	Chemotherapy, immune suppression	18–65 years	20	NCT03441958
Completed	Donor Umbilical Cord Blood Transplant With or Without Ex-vivo Expanded Cord Blood Progenitor Cells in Treating Patients With Acute Myeloid Leukemia, Acute Lymphoblastic Leukemia, Chronic Myelogenous Leukemia, or Myelodysplastic Syndromes	Nohla Therapeutics, Inc., USA	Randomized open label parallel arm	Leukemias/ myelodysplastic Syndrome	Engraftment	NLA101	N/S	Chemotherapy, immunosuppression, unmanipulated UCB	6 m-65 years	163	NCT01690520
Recruiting	Donor Natural Killer Cells, Cyclophosphamide, and Etoposide in Treating Children and Young Adults With Relapsed or Refractory Solid Tumors	M.D. Anderson Cancer Center, USA	Open label single arm	Solid tumours	Safety & maximum tolerated dose	UCB expanded NK cells	N/S	Chemotherapy	1–40 years	38	NCT03420963
Recruiting	Cord Blood Transplant With OTS for the Treatment of HIV Positive Hematologic Cancers	Fred Hutchinson Cancer Research Center, USA	Non-randomized open label parallel arm	Hematologic Cancers	Engraftment	NLA101	N/S	Chemotherapy, TBI, immunosuppression, unmanipulated UCB	6 m-65 years	10	NCT04083170
Completed	Clofarabine, Cytarabine, and Filgrastim Followed by Infusion of Non-HLA Matched Ex Vivo Expanded Cord Blood Progenitors in Treating Patients With Acute Myeloid Leukemia [88]	Nohla Therapeutics, Inc., USA	Open label single arm	Acute myeloid leukemia	Safety	NLA101	N/S	Chemotherapy	18–70 years	29	NCT01031368
Completed	Allogeneic SCT of NiCord®, UCB-Derived Ex Vivo Expanded Stem and Progenitor Cells, in Patients With Hemoglobinopathies [89]	Gamida Cell ltd, USA	Open label single arm	Hemoglobinopathies	Safety & engraftment	NiCord®	1.85–29.37 × 10^6^ cells/kg	Unmanipulated UCB	2–45 years	20	NCT01590628
Completed	A Safety Study of Human Cord Blood Derived, Culture-expanded, Natural Killer Cell (PNK-007) Infusion With or Without Subcutaneous Recombinant Human Interleukin-2 (rhIL-2) Following Autologous Stem Cell Transplant for Multiple Myeloma (MM) [90]	Celularity Incorporated, USA	Open label single arm	Multiple myeloma	Safety & maximum tolerated dose	PNK-007 (NK cells)	N/S	rhIL-2	18–70 years	15	NCT02955550
Completed	A Pilot Study to Evaluate the Co-Infusion of Ex Vivo Expanded Cord Blood Cells With an Unmanipulated Cord Blood Unit in Patients Undergoing Cord Blood Transplant for Hematologic Malignancies	Fred Hutchinson Cancer Research Center, USA	Open label single arm	Leukemias/ lymphomas	Safety & engraftment	N/S	N/S	Chemotherapy, irradiation, immunosuppression, unmanipulated UCB	6 m-45 years	23	NCT00343798
Recruiting	US Study of ECT-001-CB in Pediatric and Young Adult Patients With High-Risk Myeloid Malignancies	ExCellThera inc., Canada	Open label single arm	High risk myeloid malignancies	Adverse events & relapse	UM171	0.25–5 × 10^6^ cells/kg	Chemotherapy, immunosuppression, GVHD prophylaxis, unexpanded UCB CD34- infusion	0–21 years	16	NCT04990323
Completed	Cord Blood Expansion on Mesenchymal Stem Cells [91]	M.D. Anderson Cancer Center, USA	Open label single arm	Myelodysplastic syndrome & Leukemia	Engraftment	UCB- CD34+ cells expanded with MSC co-culture	5.08 × 10^6^ cells/kg	Chemotherapy	18–65 years	16	NCT00498316
Recruiting	Safety Study and Therapeutic Effects of Umbilical Cord Blood Treg on Autoimmune Diabetes	Second Xiangya Hospital of Central South University, China	Open label Parallel arm	Type 1 diabetes	Safety & tolerability	Expanded UCB derived Tregs	1–5 × 10^6^ cells/kg	Insulin	6–60 years	40	NCT02932826
Recruiting	Engineered NK Cells Containing Deleted TGF-BetaR2 and NR3C1 for the Treatment of Recurrent Glioblastoma	M.D. Anderson Cancer Center, USA	Non-randomized Open label Parallel arm	Recurrent Glioblastoma	Toxicity	Genetically modified UCB derived NK cells	N/S	N/S	< 18 years	25	NCT04991870
Completed	MGTA-456 in Patients With Inherited Metabolic Disorders Undergoing Hematopoietic Stem Cell Transplantation (HSCT) [76]	Magenta Therapeutics, Inc., USA	Open label single arm	Inherited metabolic disorders	Engraftment	MGTA-456 (SR-1)	110 × 10^6^ cells/kg	N/S	0–17 year	8	NCT03406962

*N/S* not specified, *UCB* Umbilical cord blood, *VPA* Valproic acid, *GVHD* graft versus host disease, *NK* natural killer, *m* months, Information obtained from clinicaltrials.gov (Search terms: Expanded umbilical cord blood; Umbilical cord blood expansion). List is up to date as of the 14/12/2023. List does not include withdrawn/terminated studies or those with an unknown status

#### Nicotinamide

Nicotinamide is a vitamin B3 derivative that is known to inhibit CD34 differentiation. It is thought to do so by inhibiting Sirtuin 1 (SIRT1), a deacytylase, which plays a role in regulating normal haematopoietic stem cell regulation [92]. This is further confirmed using mouse models where SIRT1 deficient mice exhibit increased proliferation of primitive CD34 cells in vivo [93]. A nicotinamide expanded UCB product, Omisirge (previously Omidubicel, NiCord® or cordIn) has been tested in 6 different clinical trials (Table 3) and expansion using nicotinamide results in up to 486-fold increase in cells after 21 days of expansion [85]. Two of these completed studies have demonstrated improved time for neutrophil recovery compared to historical controls and have demonstrated the safety of Omisirge as a cell therapy option [85, 84]. This product has recently been granted market approval from the FDA [45].

#### Notch ligands

Members of the notch gene family are known to be expressed in CD34+ cells, including haematopoietic progenitors, and have been shown to mediate cell-fate decision during haematopoiesis [94]. The notch ligand Delta 1 has been shown to activate notch signalling in HSCs and promote HSC-self renewal [95]. Dilanubicel (NLA101) is a Delta 1 expanded UCB product that has been tested in 3 completed clinical trials and is currently being tested in 2 additional clinical trials (Table 3). Results from one of the completed studies has shown that expansion with Delta 1 resulted in an increase in total cells by 1099-fold, and an average fold expansion of CD34+ cells of 141-fold. Further, CD34+ cells made up only 30–35% of the final expanded product, suggesting that activation of notch ligand signalling promotes cell proliferation without preventing differentiation [86].

#### Copper chelator

Copper has previously been shown to regulate haematopoietic progenitor cell proliferation and differentiation, and lowering cellular copper using Tetraethylenepentamine (TEPA), a copper chelator, lowers cell differentiation [96]. Preclinical studies have shown that culture with TEPA results in an average of 17-fold increase in CD34+ cells after three weeks of expansion, and 1594-fold increase after 11 weeks of culture [44]. Currently there have been two completed clinical trials testing a tetraethylenepentamine expanded UCB product, carlecortemcel-L (StemEx®). An initial phase I/II clinical trial reported only a median 2.26-fold increase in CD34+ cells after culturing for 21 days with the final product comprising of 12.8% CD34+ cells [97]. A subsequent clinical trial reported a median of 90-fold increase in CD34+ cells, with the final product consisting of only 18.2% CD34+ cells [87].

#### Valproic acid

Valproic acid (VPA) is a HDAC inhibitor (HDACI) which has been investigated as a method for expanding HSCs. HDACIs are known to upregulate pluripotency genes, which when these genes are knocked down leads to a reduction in CD34+CD90+ cells [98]. A preclinical study demonstrated a 213-fold increase in CD34+ cells and a 20,202-fold increase in CD34+CD90+ cells after 7 days. 75% of the final expanded product were CD34+CD90+ [98]. There has been one clinical trial completed utilising VPA expanded HSCs, however the results for this study have not yet been published.

#### Other

Other methods of HSC expansion utilises a co-culture system with other cell types including MSCs [99–91] and adult endothelial cells [101, 102]. Co-culture systems aim to recapitulate the hematopoietic stem cell niche, where HSCs have continued contact with other niche cells to promote proliferation. There are also other methods of expansion currently being investigated to expand UCB derived non-HSCs such as mesenchymal progenitor cells (MPCs), natural killer (NK) cells [90], T-cells [80], Tregs and monocytes. These methods of UCB cell expansion have all been tested in clinical trials (Table 3).

### Clinical data supporting the use of expanded UCB

Currently there are 36 registered clinical trials that are investigating expanded UCB as a cellular therapy, including 22 completed trials (*clinicaltrials.gov;* Table 3). The majority of these trials use expanded HSCs (26/36), with the remaining trials using lymphocyte derivatives including expanded NK cells, T-cells and Tregs or MPCs/MSCs. In addition, most of these trials are focused on transplantation applications for haematological conditions (31/36) such as leukemia, lymphoma and myelodysplastic syndromes. Other conditions include metabolic disorders (3/36) such as type 1 diabetes, and neurological conditions such as multiple sclerosis (1/36) and glioblastoma (1/36), where the target of the therapy is for regeneration, not engraftment.

Of the 22 completed trials using expanded UCB CD34+ cells, 15 trials have published results [43, 74, 76, 85, 84, 86, 87, 91, 90–81]. A recent systematic review and meta-analyses [103] of these published studies has indicated that treatment with ex vivo expanded UCB can accelerate engraftment of platelets and neutrophils, and all but one study showed that treatment with expanded UCB resulted in a significant reduction in time to neutrophil recovery compared to controls. Meta analyses of these studies also revealed a significant decrease in the risk of death following expanded UCB infusion, compared to controls [103].

Whilst the results from current trials are promising, many of the listed clinical trials are open label, single-group studies that have the primary outcome of safety (26/36), with only 5 of 36 being randomized controlled trials (Table 3). Safety studies are important and are the necessary first step to progress any new therapy through ethics and governance bodies, and 9 completed studies now report safety in a total of ~ 300 patients ranging from 3 to 65 years of age [103]. One limitation of these studies is that there is a large amount of heterogeneity in the cell treatment regimes being implemented in these trials. This includes the method by which the cells are expanded and the timing and dosage of cell treatments. In addition to an expanded cell product, many studies also include administration of an accompanying unmanipulated UCB unit, or the unexpanded portion of the UCB unit that underwent expansion.

As the main use for expanded HSCs is currently in transplantation medicine, the safety and efficacy of these cells has not been well established for regenerative medicine purposes. Despite this, infusion of cells in most regenerative medicine applications does not require ablation of the immune system and does not require the cells to engraft to be beneficial, thus it is predicted that infusion of cells for regenerative medicine purposes will not be as challenging as in transplantation applications. Further, a recent systematic review by Paton et al. has concluded that allogeneic administration of unexpanded UCB in regenerative medicine applications is considered safe and has not been associated with severe adverse events [104].

### Preclinical studies supporting the use of expanded UCB as a therapy

Expanded UCB derived CD34+ cell therapies have been the subject of preclinical studies to establish the therapeutic benefits in the setting of haematological conditions, including cancers. Predominantly these studies are conducted using immunodeficient mouse models and data from these studies has provided the scientific basis for clinical translation of expanded UCB therapies for transplantation [73]. In addition, there have been a large number of preclinical studies that are focused on optimisation of expansion techniques and understanding the mechanisms of UCB expansion in vitro [71, 73].

Outside of haematology research, there have been very few preclinical studies that have investigated the efficacy of expanded UCB cells in a regenerative medicine capacity. One study trialled expanded UCB cells in vivo as a therapeutic option for traumatic spinal cord injury in an immunosuppressed rat model. Chua et al. demonstrated in this study that rats treated with expanded CD34+ cells demonstrated functional recovery when compared to untreated controls [52]. Analysis of expanded cell conditioned media revealed increased expression of anti-inflammatory (TIMP-1 and TIMP-2), angiogenic (VEGF, IL-8 & angiogenin) and neuroprotective (BDNF, PDGF-BB and EGF) factors [52]. A subsequent study by Whiteley et al. has investigated expanded UCB CD34+ cells as a potential therapy for hind limb ischemia in mice [53]. In this study, treatment with expanded CD34+ cells resulted in improved blood flow to the ischemic limb and decreased necrosis of the foot. As the mouse model used did not allow for cell engraftment, positive effects of expanded CD34+ cells were determined to be a result of paracrine signalling. Further proteomic analysis of conditioned cell expansion media identified an increase in proteins involved in tissue repair (FGF-9), extracellular matrix production and maintenance (IGF-1, PDGF-BB, MMP-9, TIMP-1 and TIMP-2), angiogenesis (IL-3, IL-4, VEGF and EGF) and activation and maintenance of inflammatory processes (MIPs, MCP-4, TGF-β 3) [53].

The neuroprotective properties of expanded UCB CD34+ cells have also been investigated in vitro [79]. CD34+ cells were expanded using standard growth factors UM171 and SR-1. Expanded cells had higher gene expression of neurotrophic factors (BDNF, GDNF, NTF-3 and NTF-4) and angiogenic factors (VEGFA and ANG), compared to unexpanded CD34+ cells. Further, expanded CD34+ cells promoted glial cell proliferation and vascular tube formation and reduced oxidative stress to a greater degree than unexpanded CD34+ cells [79].

**Fig. 2:**
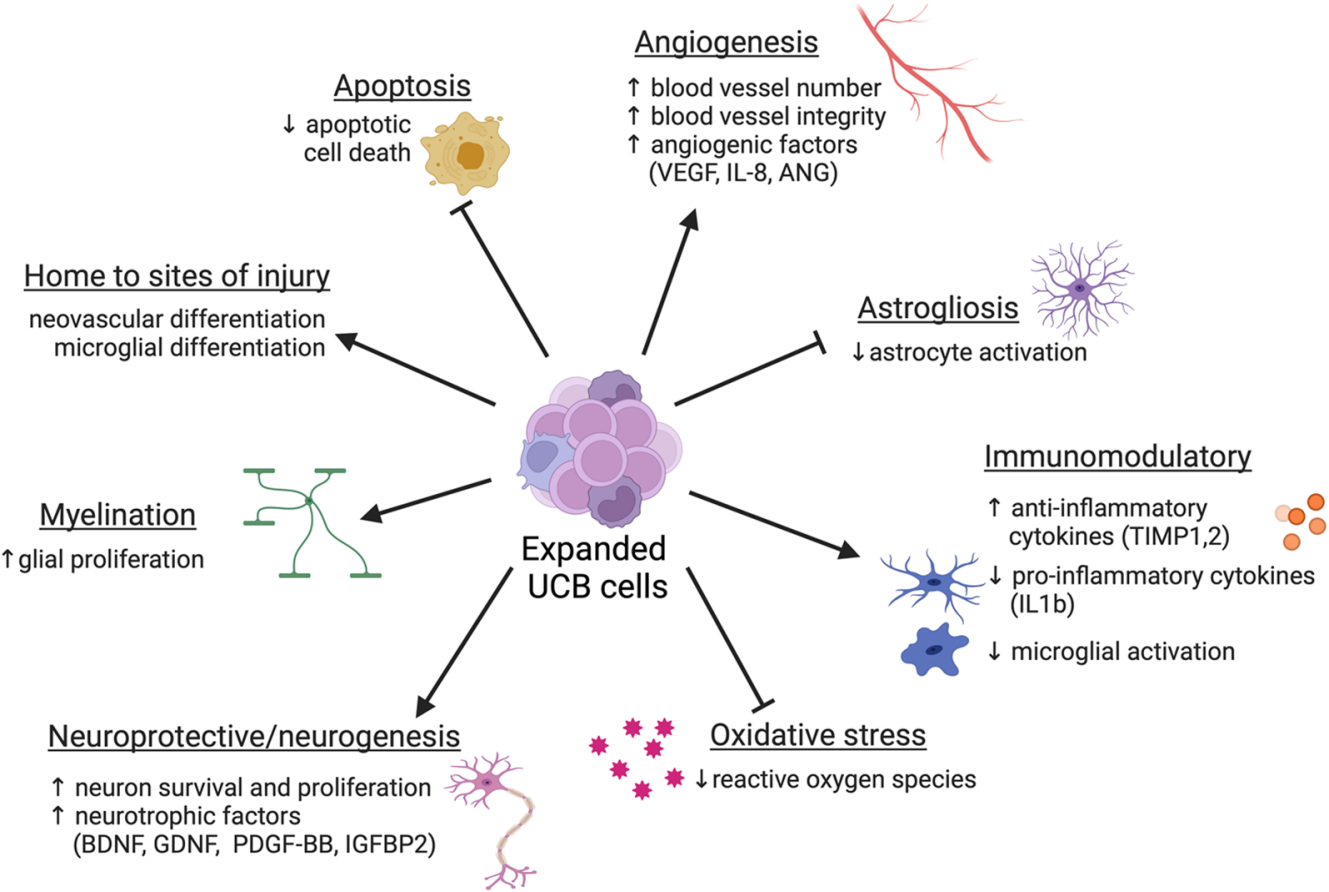
Mechanisms of action of expanded UCB derived CD34+ cells. Data from preclinical studies suggests that expanded UCB derived CD34+ cells have many beneficial properties for regenerative medicine applications. These cells are neuroprotective, immunomodulatory and angiogenic. (Created with BioRender.com)

Taken together, these studies support anti-inflammatory, angiogenic and neuroprotective roles of expanded CD34+ cells, and emphasise the therapeutic potential of CD34 expansion for non-haematological diseases (Fig. 2).

## Umbilical cord blood cell therapies for brain injury

Umbilical cord blood derived cells have been extensively researched in preclinical and clinical studies as a potential cell therapy option in the field of neurological injuries. The topic of UCB as a therapy for brain injury in clinical and preclinical studies has been well reviewed [104–111], and the potential efficacy of treatment with UCB has been shown in a variety of conditions. These includes in adults, for treatment of traumatic brain injury (TBI) [112, 113], stroke [18, 19] and spinal cord injury [22, 23], and conditions in babies/children including cerebral palsy (CP) [48, 49], hypoxic ischemic encephalopathy (HIE) [14, 46, 114], preterm birth [115, 116] and fetal growth restriction (FGR) [37].

Briefly, preclinical studies have shown that UCB mononuclear cells are neuroprotective and able to modulate multiple aspects of brain injury. A recent systematic review and meta-analysis of preclinical studies by Nguyen et al. has highlighted the efficacy of UCB cells as a therapy for perinatal brain injury. Specifically, UCB cell administration increases neuron and oligodendrocyte cell numbers, reduces cell death and microglia activation. Further, UCB has been shown to modulate neuroinflammation, resulting in a significant decrease in the pro-inflammatory cytokines TNF-α, IL-6 and IL-1β. UCB cells have also been shown to improve motor function as determined by the cylinder test and rotarod test [38].

Several clinical trials have also been conducted to investigate the efficacy of UCB therapies for non-haematological malignancies where the most commonly reported use of UCB as a therapy was for neurological diseases. This includes cerebral palsy, autism, TBI, stroke and spinal cord injury, with cerebral palsy accounting for the majority of neurological UCB clinical trials [117]. Results from various clinical trials have demonstrated that both autologous and allogeneic administration of UCB for neurological conditions is safe and is not associated with severe adverse events [104, 105]. The efficacy of UCB cell therapies for neurological conditions has only been reported in a few clinical trials. Overall, results from clinical trials in the setting of cerebral palsy have shown that UCB administration improved motor and cognitive outcomes [118] and preclinical and clinical studies combined show that UCB derived MNCs are effective at improving various pathologies associated with brain injury in adults and children.

## Neuroprotective and neuroregenerative potential of CD34+ cells

Whilst unexpanded CD34+ stem cells have been well studied as a therapy for haematological malignancies, there are limited studies looking at this population of cells for other conditions including brain injury. Here we will summarise the in vivo and in vitro studies that have investigated the use of CD34+ cells as a therapy for neurological injuries, as well as the action of endogenous CD34+ cells in response to brain injury.

### Actions of endogenous CD34+ cells in response to brain injury

The action of endogenous mobilised CD34+ cells have been studied in response to a neurological insult, most commonly ischemic stroke and TBI. In a rat model of TBI, bone marrow derived CD34+ cells are rapidly mobilized into the peripheral blood, reaching a peak at 48 h post insult. These cells then homed to the site of injury, resulting in a significant increase in CD34+ cells in the ipsilateral hemisphere, with a peak in cell numbers occurring at 7 days post TBI. There was also an increase in microvasculature density up to 14 days post TBI in the injured tissue, suggesting that the CD34+ cells promote neovascularization [119].

Mobilisation of CD34+ cells has also been detected in the setting of ischemic stroke. Using a mouse model of stroke following a bone marrow transplant there was a significant increase in BM CD34+ cells found in the ipsilateral hemisphere of the brain 6 weeks and 6 months following stroke injury. Cell double labelling determined that more than 90% of these cells displayed microglia markers [120]. UCB and MBP CD34+ cells injected into immunodeficient mice have also been shown to differentiate into microglia. In a study by Asheuer et al., CD34+ cells from both sources were administered intravenously to immunodeficient mice. Analysis of post-mortem tissue demonstrated that 95–100% of engrafted human cells expressed RCA-1 lectin, a marker of perivascular microglia. 50% of engrafted cells also expressed the Iba1 antigen, a marker of ramified microglia. It is proposed that the ability for CD34+ cells to differentiate into microglia in the brain may be due to the common origin of microglia and the haematopoietic system, the yolk sac [121].

Transplanted BM CD34+ cells have also been detected in the vasculature in the acute period following induction of stroke, with these cells displaying endothelial cell markers [122]. Further, higher levels of circulating CD34+ cells have been detected in humans who have experienced an ischemic stroke [123, 124]. In fact, the number of circulating CD34+ cells present in peripheral blood after a stroke event has been shown to be correlated with the degree of functional and neurological recovery [125, 126]. However this mobilisation of CD34+ cells has been shown to be muted when patients have been treated with tissue-type plasminogen activator (tPA), the standard treatment option for stroke [127].

The mobilisation of CD34+ cells in response to injury is likely to be a protective mechanism that can promote neovascularisation or perhaps promote an anti-inflammatory response, highlighting the therapeutic potential of CD34+ cells for neurological conditions. As such it is proposed that mobilising CD34 cells after injury, or delivery of exogenous CD34+ cells, could provide an avenue for repairing injured cerebral tissue.

### Treatment of neurological conditions with CD34+ cells

As previously mentioned, treatment with CD34+ cells is generally targeted towards haematological conditions, however the efficacy of CD34+ cells as a therapy for neurological conditions has been investigated in a number of preclinical studies.

Previous studies have focused on investigating the efficacy of CD34+ cells as a therapy for adult stroke injury using the middle cerebral artery occlusion (MCAO) model. One of the key outcomes that has shown to be improved following CD34+ cell administration was motor and behavioural outcomes. Specifically, CD34+ cells have been shown to reduce hyperactivity [50, 128], improve spatial learning and memory [129], and improve motor deficits including balance and strength as determined by beam walk and rotarod testing respectively [130]. Further, two such studies have shown that treatment with CD34+ cells resulted in an improved motor and neurological score using the modified neurological severity score (mNSS) [130, 131].

As with other UCB cell types, CD34+ cells are thought to convey neuroprotection through trophic mechanisms, however, CD34+ cells have been shown to migrate to the site of injury and differentiate in neural cell subtypes. Specifically, infused cells have been detected generally in both the ipsilateral and contralateral hemispheres [131, 132], as well as specifically homing to the border zone of the ischemic lesion [130]. Further, small numbers of CD34+ cells that have migrated to the brain display markers of microglia [132], neurons, astrocytes and endothelial cells [131].

Aspects of neuropathology are modulated following CD34+ cell administration including astrogliosis [133], apoptosis, and neuroinflammation [132]. Further, an increase in neurogenesis, and thus neural cell populations [50], and expression of BDNF was seen after CD34+ cell administration [133].

Lastly, in an adult rat model of Parkinson’s disease, CD34+ cells improved limb asymmetry as seen by the cylinder test. Infused CD34+ cells were detected in the brain; however, they were not positive for markers of neurons, astrocytes or oligodendrocytes. Treatment with CD34+ cells also induced neovascularization, reduced astrogliosis and preserved dopamine producing neurons [51].

The efficacy of CD34+ cells has also been investigated in the setting of neonatal brain injury, specifically using the MCAO model of stroke, and the Rice–Vannucci model of hypoxic-ischemic (HI) brain injury. Some of these studies have shown that treatment with CD34+ cells resulted in small improvements in behavioural outcomes, particularly locomotor activity [134] and limb use [135], whilst other studies showed little to no improvement in motor and behavioural outcomes [47, 135, 136]. Further, some aspects of neuropathology were improved with cell administration, including an increase in neurogenesis [134, 136] and a decrease in apoptosis related genes [137], however CD34+ cells were not able to significantly reduce tissue loss [135].

From these few studies, it appears that the efficacy of CD34+ cell administration for perinatal brain injury was not as evident as in the adult population. This could be due to the timing of administration or cell dose used. The majority of neonatal studies delivered cells 48 h after injury with doses ranging from 1.5 × 10^4^ to 1 × 10^5^. Conversely, in the adult studies, cells were delivered as early as 30 min after stroke, with 24 h being the most common administration timepoint. Cell doses also ranged from 5 × 10^5^ to 5 × 10^6^ cells. This could suggest that the neuroprotective benefits of CD34+ cells is dependent on cell dose and timing. Further, there are differences in the way in which injury progresses between adults and neonates following an ischemic insult [138]. This could contribute to discrepancies in the efficacy of CD34+ cells following an ischemic injury, thus the timing and dose of cell administration should be optimised for neonatal ischemia. In order to reduce heterogeneity in studies, cell dosages should be consistent to reflect cord blood cell doses used in clinical trials and shown to be effective, which is often 25–50 × 10^6^ cells/kg [105].

Taken together, this preclinical evidence demonstrates that CD34+ cells have the potential for improving aspects of brain injury, including engraftment and differentiation into neural cell subtypes, however optimisation will be needed for cell doses and timing. Further, the limited availability of HSCs derived from UCB is a potential roadblock for translation into clinical use for regenerative medicine, thus it is proposed that HSC expansion will allow us to overcome this barrier. It is clear that preclinical work on expanded UCB cells as a therapy is limited and no such study has tested the neuroprotective potential of these expanded UCB cells in an in vivo model of brain injury. Consequently, we are currently investigating the potential of expanded UCB derived cells particularly for neonatal neuroprotection.

## Future applications of UCB expansion

This review highlights the current progress of HSC expansion and demonstrates the evolution of expanded UCB therapies from preclinical studies into clinical trials. Results from clinical trials have established the safety of expanded UCB therapies in adults and children as young as 3 years of age, particularly for treatment of haematological malignancies, with few adverse events reported as a direct result of expanded UCB infusion. Despite this clinical evidence, the current application of expanded UCB is very narrow. Preclinical evidence supports the application of this novel cell therapy for treatment of neurological conditions.

Preclinical studies have highlighted the benefits of unexpanded CD34+ cells in neurological conditions, specifically for ischemic stroke, as well as the differentiation and homing ability in response to brain injury. These preclinical studies demonstrate efficacy in adult models of stroke, however the efficacy for perinatal brain injury has not yet been well established. There are however, very few studies that have explored the use of CD34+ cells in perinatal brain injury, thus more studies are needed to determine their true potential. Further, these studies suggest that cells may work in a time and dose dependent manner, thus consistency should be employed between studies to ensure that appropriate conclusions can be drawn regarding the efficacy of CD34+ cells for modulating brain injury. In addition, only two studies have been conducted where expanded UCB cells were used for regenerative medicine purposes, and these studies have shown that expanded UCB demonstrated a degree of tissue repair and functional recovery in models of spinal cord injury and hind limb ischemia respectively.

It is proposed that expanded UCB derived HSCs will be a key therapy candidate for neurological conditions and this technique will allow for autologous treatment for babies with insufficient cells available, “off the shelf” allogeneic therapies, and will allow for administration of repeat doses of cells, which have been shown to be more beneficial than a single dose alone [14, 139, 140]. The use of expanded UCB also reduces the need for the infusion of multiple cord blood units to reach sufficient therapeutic cell numbers for infusion, thus finding appropriately HLA matched samples will be simpler.

Stem cell expansion will be beneficial for both autologous and allogeneic applications. Specifically, in autologous settings, where cells could be used soon after collection for therapy or banked for later use. This is particularly important where there may not be enough cells available for infusion, such as in cases where the baby has a small placenta and low cord blood volume, which is often the case with babies who are born preterm [141]. In these circumstances, cord blood expansion will ensure that an appropriate therapeutic dose is met. Further, expansion will allow for the same allogeneic donor to be used in clinical treatments to reduce the incidence of rejection and GVHD and allows for the formation of an off the shelf cell therapy that can be easily accessed, particularly in low resource settings.

## Conclusion

In summary, further studies should be conducted to determine the therapeutic efficacy of expanded UCB derived HSCs for neurological conditions, particularly in neonates. This potential therapy provides a novel avenue for cell therapies that will be more accessible and allows for more standardised “off the shelf” therapies for babies, children and adults.

## Data Availability

Not applicable.

## Abbreviations

UCB: Umbilical cord blood
MNC: Mononuclear cell
HSC: Haematopoietic stem cell
MSC: Mesenchymal stem cell
Treg: T regulatory cell
MDSC: Monocyte derived suppressor cell
EPC: Endothelial progenitor cell
GVHD: Graft versus host disease
HLA: Human leukocyte antigen
HSPCs: Haematopoietic stem and progenitor cells
CD34: Cluster of differentiation 34
BM: Bone marrow
MPB: Mobilised peripheral blood
G-CSF: Granulocyte colony stimulating factor
TPO: Thrombopoietin
Flt3: Fms-like tyrosine kinase 3 ligand
IL: Interleukin
SCF: Stem cell factor
AhR: Aryl hydrocarbon receptor
SR-1: Stem Regninin-1
LSD1: Lysine-specific histone demethylase 1A
HDACI: Histone deacytylase 1
RCOR1: Rest corepressor 1
SIRT1: Sirtuin 1
TEPA: Tetraethylenepentamine
VPA: Valproic acid
HDACI: Histone deacytylase inhibitor
MPCs: Mesenchymal progenitor cells
NK: Natural killer cells
VEGF: Vascular endothelial growth factor
BDNF: Brain derived neurotrophic factor
PDGF-BB: Platelet-derived growth factor subunit B
EGF: Epidermal growth factor
TIMP-1/2: Tissue Inhibitor of Metalloproteinase 1/2
FGF: Fibroblast growth factor
MIP: Macrophage inflammatory protein
TGF-β: Transforming growth factor beta
MMP-9: Matrix Metalloproteinase 9
MCP-4: Monocyte chemoattractant protein 4
NTF-3/4: Neurotrophin 3/4
ANG: Angiopoietin
TBI: Traumatic brain injury
CP: Cerebral palsy
HIE: Hypoxic ischemic encephalopathy
FGR: Fetal growth restriction
tPA: Tissue-type plasminogen activator
MCAO: Middle cerebral artery occlusion
mNSS: Modified neurological severity score
HI: Hypoxia ischemia
